# The CRF domain defines Cytokinin Response Factor proteins in plants

**DOI:** 10.1186/1471-2229-10-74

**Published:** 2010-04-26

**Authors:** Aaron M Rashotte, Leslie R Goertzen

**Affiliations:** 1 Department of Biological Sciences, Auburn University, Auburn AL 36849-5407 USA

## Abstract

**Background:**

Cytokinin Response Factors (CRFs) are a small subset of AP2/ERF transcription factor genes shown in Arabidopsis to regulate leaf development as part of the cytokinin signal transduction pathway. This study examines the phylogenetic distribution of CRF genes in other plant species, and attempts to identify the extent of sequence conservation and potential gene function among all CRF genes.

**Results:**

We identified CRF genes in representatives of all major land plant lineages, including numerous flowering plant taxa in addition to the model systems in which ERF genes have been catalogued. Comparative analysis across this broader sampling has identified strongly conserved amino acid motifs other than the AP2/ERF domain for all CRF proteins as well as signature sequences unique to specific clades of CRF genes. One of these motifs, here designated as the CRF domain, is conserved in and unique to CRF proteins distinguishing them from related genes. We show that this novel domain of approximately 65 amino acids is found in CRF proteins from all groups of land plants and only in CRF genes. Phylogenetic analyses suggest that the evolution of CRF genes has included numerous duplication events. In this phylogenetic context we examine protein evolution including the gain and loss of accessory domains, correlate these molecular evolutionary events with experimental data on cytokinin regulation and speculate on the function and evolution of the CRF domain within AP2/ERF transcription factor proteins. We also tested a prediction drawn from the phylogenetic analyses that four CRF domain containing genes from Tomato, previously unexamined for cytokinin response, are transcriptionally inducible by cytokinin, supporting the link between CRF genes, CRF-specific domains and cytokinin regulation.

**Conclusion:**

CRF genes can be identified in all lineages of land plants, as a distinct subset of AP2/ERF proteins containing a specific and unique CRF domain. The CRF domain can be used to identify previously unclassified predicted genes or genes identified only as members of the AP2/ERF protein family. CRF domain presence and phylogenetic relatedness to known Arabidopsis CRF genes predicts gene function to some extent.

## Background

AP2/ERF proteins comprise one of the largest families of transcription factors in plants and are defined by the presence of an AP2 DNA binding domain of around 68 amino acids. This protein family can be broadly divided into those proteins with two AP2 domains, e.g. the eponymous floral patterning gene APETELA2, and those with a single AP2 domain such as the Ethylene Response Factors (ERFs) [1-5]. To date, all of the proteins containing a canonical AP2 DNA binding domain have been identified in land plants, but more divergent AP2 homologues have been hypothesized for cyanobacteria, ciliates and viruses [3,6]. The AP2 DNA binding domain has been shown to interact with slightly varied DNA sequences depending on its exact composition e.g. DREB subfamily members have been shown to bind DRE/CRT cis-acting elements and ERF subfamily members have been shown to bind GCC box cis-acting elements [2,7]. Through these interactions AP2/ERF genes control a variety of developmental and environmental response processes in plants.

The ERF subfamily comprises the majority of all AP2/ERF proteins in Arabidopsis (83%), rice and other sequenced plant genomes. They are further distinguished from single AP2 domain-containing proteins by the absence of a B3 domain, a feature reserved for the class of genes designated as RAV proteins [2,5,8]. Through whole-genome analyses of Arabidopsis, Populus and rice, the ERF subfamily has been divided into 12 more or less distinct subgroups [2,5,8,9]. Several genes in Arabidopsis from the ERF subgroup B-5 (also known as subgroup VI) have recently been identified as cytokinin regulated transcription factors or CRFs [10]. Although CRF genes have been examined in a preliminary way in Arabidopsis and homologs have been identified in rice there has been little done to establish their presence at a broader phylogenetic scale or to examine basic aspects of molecular evolution such as potentially conserved motifs within these proteins.

In this study we set out to better understand the function of CRFs and CRF related proteins in other species, we have examined in detail the protein sequence of these group members and compared them to similar proteins in rice and a wide range of other plant species. These analyses have identified two domains other than the AP2 binding domain that are commonly present in these protein sequences, including one domain that is specific to this group of proteins and conserved throughout land plants. We have designated this novel group-specific domain the CRF domain and further analyzed it in proteins from numerous plant taxa. The presence of this domain in all land plant lineages has allowed an evolutionarily broad consensus sequence to be defined. Using this consensus sequence we have been able to further classify several ERF genes as belonging to the CRF subgroup, and show that four such CRF genes in Tomato previously unexamined for cytokinin response are cytokinin inducible. Furthermore, phylogenetic analysis of these sequences, their relationship to the AP2 domain, and evolutionary implications are discussed.

## Results

We have identified a large number (over 100) of AP2/ERF genes from diverse land plant lineages that are orthologous to a set of genes from Arabidopsis known as Cytokinin Response Factors or CRFs.

**Figure 1:**
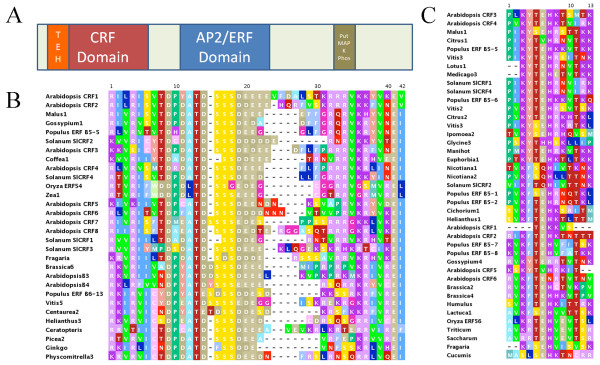
**The CRF domain**. (A) A model of CRF proteins in which the position of CRF domain relative to AP2 DNA binding domain that it always accompanies is shown, in addition to a C-terminal domain present in some members. (B) CRF domain. The core consensus sequence and alignment of the CRF domain from representative members of over 125 related CRF proteins is shown. (C) TEH region of the CRF domain found preceding a subset of members of the CRF proteins. Alignment of representative members is shown.

Sequence analysis and alignment of CRF genes or genes containing a CRF domain, as designated below, was initiated with the six previously identified CRF genes from Arabidopsis, CRF1-6, and two closely related Arabidopsis genes, At1 g71130 and At1 g22985, that we now designate CRF7 and CRF8, respectively. These sequences in addition to previously identified CRF gene homologs from Rice, as seen in Nakano et al., 2006 [8], allowed us to generate a basic consensus sequence that we used to gather further CRF sequences through a series of BLAST analyses. Additional searches with a representative species sequence within genus or specific genome sequencing efforts allowed us to generate a broader CRF protein consensus sequence, the CRF domain of which is presented in Figure 1. Sequences are identified simply by their generic name followed by a number if more than one was identified per genus, unless previously designated a name or for the *Solanum lycopersicum *or SlCRFs (SlCRF1 also previously designated as PTI6 is from SGN-U314347, SlCRF2 is from SGN-U329134, SlCRF3 is from SGN-U344182, and SlCRF4 is from SGN-U331355), with full gene names included see Additional File 1. These results show that CRF genes are present throughout land plants and always consist of a novel N-terminal CRF motif/domain, an AP2-DNA binding domain near the middle of the protein, and in roughly half of the sequences a putative kinase phosphorylation site in the C-terminal region.

### CRF Protein Domains

The AP2 domain as has been previous detailed for CRF protein members is centered on the base amino acid sequence AAEIRD**RR*R*WLGT*DTAEEAA where the underlined WLG amino acids are absolutely required for AP2/ERF domain binding to DNA and the * represent non-specifically conserved amino acids [2,8].

While the specific sequence found in CRF protein members for this domain is quite similar to previously described alignments using ERF proteins from Arabidopsis and Rice, as seen in Nakano et al., 2006 [8], we have shown it to be present far beyond these two species, in fact occurring throughout land plants. While AP2 domains in general maintain a number of conserved amino acids that are required for their function, there is also specificity within a domain that can in some cases determine DNA sequence binding specificity. A prime example is the difference between the conserved amino acid sequence VAEIRE from the CBF/DREB subfamily of ERF proteins and AAEIRD from the ERF subfamily resulting in binding to DRE/CRT or GCC box cis-elements respectively [2]. The sequence of the AP2 domain found in this study of CRF proteins, indicates that they belong to the ERF protein subfamily, but also indicates a higher level of specificity within this group.

**Figure 2:**
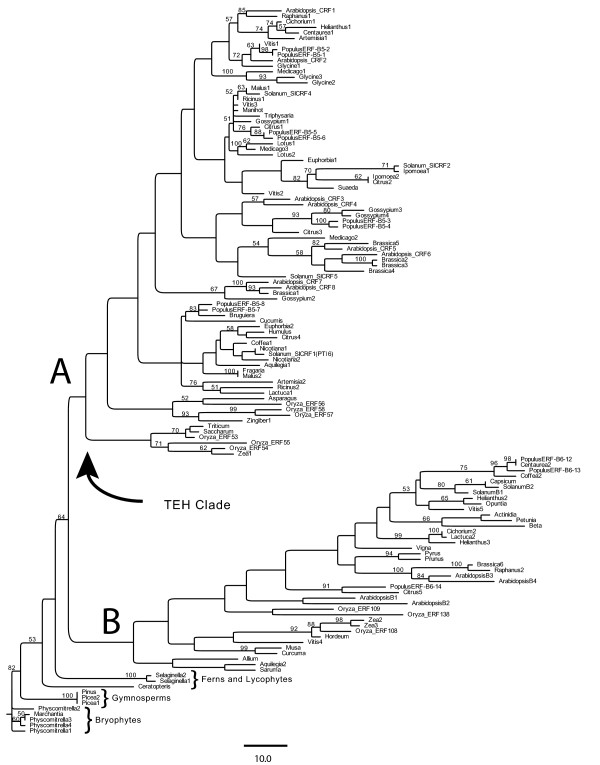
**Neighbor joining tree of CRF proteins based on alignment of CRF and AP2 domains**. Individual branches are denoted by genus name followed by a previously reported specific gene name or GenBank designator. The tree is rooted on *Physcomitrella *(moss) CRFs. Support values are indicated at nodes when found in at least 50% of 1000 bootstrap replicates.

We have also identified a novel domain of approximately 65 amino acids that is present in all CRF proteins throughout all land plants, that we have designated as the CRF domain. The consensus sequence of this domain along with a sequence alignment from representative species is shown in Figure 1, broken into two parts the core CRF domain of about 40AA (Figure 1B) and the TEH region of about 13 AA that precedes the core domain in nearly all sequences belonging to the TEH clade of CRF genes (Figure 1C, Figure 2).

The CRF domain is found in the N-terminal region of the protein and is always accompanied by an AP2-DNA binding domain, roughly 60 AA C-terminal to the CRF domain position (Fig 1A). Therefore, CRF domain containing proteins, or CRF proteins are a subset of AP2/ERF proteins. We have identified CRF domain proteins in liverworts, mosses, lycopods, ferns, conifers and all major lineages of flowering plants. CRF domain containing genes were not found in any species of green algae including the completely sequenced genomes of *Chlamydomonas, Micromonas *(2 spp.) and *Ostreococcus*, despite the presence of clearly identifiable AP2/ERF domain proteins in these genomes. Additionally, while highly divergent AP2/ERF-like domains have been detected in some bacteria, no recognizable CRF domains were found in any sequence searches outside of the land plants mentioned above, suggesting that they are unique in their occurrence within this group.

In an attempt to ascribe a specific function to the CRF domain, we performed a motif analysis of the CRF domain sequence. This revealed no similarity to any motifs or domains of known function. The best similarity identified in BLAST analysis, which is very weak, is to the C-terminal region of potassium voltage-gated channel subfamily S member 3 proteins Kv9.3 such as KCNS3 from Humans. However, the region of similarity on the potassium channel protein resides at the very end of the C-terminal of the protein that is not involved in channel structure, protein-protein interaction, or potassium movement, but is in a variable region of unknown function [11]. There is within the C-terminal region of the CRF domain a stretch of amino acids rich in lysines and arginines, which in are often involved in nuclear localization of proteins. However, there is no apparent alignment of amino acids in the CRF domain that corresponds to such known nuclear localization signal. A best guess at CRF domain function from a basic analysis of the eight members with a CRF domain that have any ascribed function, would suggest a role in cytokinin regulation, since the six CRFs from Arabidopsis appear to be regulated by that hormone [10]. It is also possible that the CRF domain may be connected to pathogen resistance, as two (non Arabidopsis) CRF domain genes, Pti6 from tomato and Tsi1 from tobacco, have been linked to pathogen resistance in gene overexpression studies [12-14]. Another possibility is that the CRF domain functions as a protein-protein interaction domain, allowing CRF domain containing proteins to form hetero or homodimers with each other or themselves.

One other small motif: SP(T/V)SVL was identified in roughly half of the CRF proteins for which we have identified full-length sequences. While a part of this motif has been previously noted for a few species we found that this conserved six AA motif occurs in CRF genes across a broad range of land plants including *Selaginella *[8,9]. This motif is predicted to function as a putative MAP kinase phosphorylation site [8,9]. Unlike the CRF domain, the SP(T/V)SVL motif is not specifically linked to either the AP2 or CRF domains (Figure 1). This SP(T/V)SVL motif can be found in 33 other non-CRF proteins in Arabidopsis alone with a variety of functions, including several different types of transcription factors. Interestingly, about half of the genes whose protein contains this domain have also been shown to have altered expression through cytokinin treatment or in a cytokinin mutant background, suggesting that CRF proteins in general may have a role in cytokinin response (Additional File 2).

### Phylogenetic Analysis of CRF proteins

CRF proteins from a wide range of land plant lineages can be readily aligned at the protein sequence level and were submitted to various phylogenetic analyses. This result is shown in the neighbor joining (NJ) tree in Figure 2 with species denoted as is Figure 1 and full gene names included in Additional File 1. In this tree there are two distinct clades that we have denoted as A and B, each of which contain sequences from diverse flowering plant lineages, with sequences from the relatively earlier branching land plant lineages at the base of the tree. This division of CRF genes into A and B clades is coincident with the presence or absence of a specific set of amino acids in the beginning of the CRF domain, here referred to as the TEH region. The TEH region of the CRF domain is well conserved and unique, found only in the CRF domains of clade A proteins, with some variability in size and sequence (Figure 1).

Within individual plant species, A clade, TEH proteins appear to be about twice as numerous as B clade: Arabidopsis (8 A clade members: 4 B clade members), Rice (6 A clade: 3 B clade), Vitis (6 A clade: 4 B clade), Populus (8 A clade: 3 B clade). Not surprisingly, A clade CRF sequences are identified in BLAST analyses roughly twice as frequently as those B clade members without a TEH region. A somewhat similar distinction of clades was observed in a cluster analysis using only the AP2 domain of all ERF proteins in Arabidopsis and separately in Rice (Nakano et al., 2006). In these studies the 'A clade' members in each species were identified as part of one of the major subgroups of ERF proteins (group VI) with the 'B clade' proteins being relegated to a smaller, related or like group to these members (group VI-L). The within-clade similarity of amino acid sequence across either the AP2 or CRF domain is quite marked and easily discerned by eye.

As there has not been a previous analysis of CRF domain proteins containing monocots and eudicots, it is interesting to note that within the distinct A and B clades there is an additional, clear division of sequences between monocots and eudicots, particular true for the CRF proteins in clade A (Figure 2). In clade B this appears to also be the general rule, with the one exception of a 'misplaced' Vitis CRF sequence.

The clustering analyses suggest that a number of duplication events accompanied the phylogenetic history of CRF proteins. The particularly striking A/B clade duplication appears to predate the divergence of monocots, magnoliids and eudicots, but happened at some point after the origin of flowering plants. Within each of the A and B clades there are additional duplication events that occurred prior to the branching off of the eudicots, leaving multiple clades of rosid, asterid and caryophyllid sequences in each. Finally, there are a number of species specific duplications that have generated only slightly differentiatied copies of CRF-domain loci. Interestingly, within sequenced genomes there are no known examples of CRF genes having arisen from tandem duplications, but this may change with the increasing number of plant genomes being sequences. The possibility of some subfunctionalization or specialization of any of these CRF subclades will be an interesting area of future research.

### Cytokinin regulation of previously unexamined CRFs

We attempted to make use of the phylogeny of so many newly identified CRF genes to further our understanding of potential CRF gene function. We examined four, previously unexamined CRF genes from Tomato that we predicted, based on their phylogenetic placement, to possess possible cytokinin regulation.

**Figure 3:**
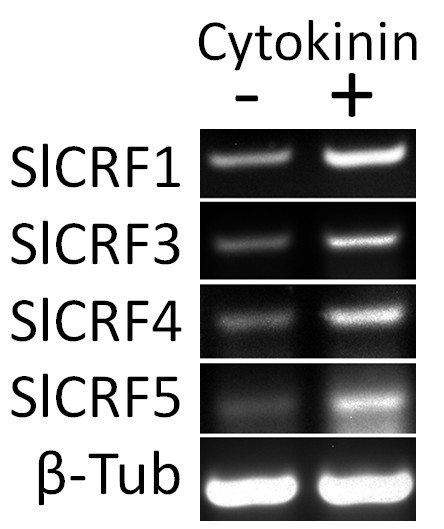
**Novel Tomato CRF genes are induced by cytokinin**. Induction of four Tomato CRF genes (SlCRFs) by 5 μM cytokinin (BA) after 2 h in leaves of 20 d old plants as shown by RT-PCR. A beta-tubulin control showing no change in expression is also shown from the same representative experiment.

Specific primers were generated for each gene such that RT-PCR could be performed on cDNA made from RNA transcripts from Tomato leaves with and without a cytokinin treatment. For simplicity we have further designated each of the four Tomato unigene constructs representing these genes as *Solanum lycopersicum *or SlCRFs (SlCRF1 also previously designated as PTI6 is from SGN-U579886, SlCRF3 is from SGN-U573201, SlCRF4 is from SGN-U574151, and SlCRF5 is from SGN-U583231). We were able to detect transcript from each of the four SlCRFs in a range of tissues (data not shown), but decided to focus on cytokinin expression in leaves. Transcript levels for all four SlCRFs were found to be induced in Tomato leaves treated for 2 hours with 5 μM cytokinin (benzyladenine) vs. a carrier control DMSO (Figure 3). This induction is similar to some of the previously examined Arabidopsis CRFs and suggests that each of these SlCRF genes is regulated by cytokinin [10]. Not only is this a novel function not previously ascribed to any of these genes, but it is the first ascribed gene function for SlCRF3, 4, and 5. This highlights the potential power of a broad phylogenetic framework for determining function of previously unknown genes.

## Discussion

In this study we define a subset of AP2/ERF proteins known as CRF or Cytokinin Response Factor proteins after the six members of this group from Arabidopsis (CRF1-6). CRF proteins can be characterized by the presence of two domains, a specific variant of AP2/ERF DNA binding domain near the middle of the protein and a novel domain at the N-terminal end that is unique to CRF genes. Additionally, in many CRF proteins there is also a putative kinase phosphorylation motif in the C-terminal half of the sequence.

The novel CRF domain is present in all CRF proteins, always found in the N-terminal region and always paired with a distinct AP2 DNA binding domain sequence. Identification of CRF proteins can be made using either of the AP2 or CRF domain alone. Previous phylogenetic studies of ERF proteins examining just the AP2 domain do return a cluster of proteins that possess the CRF domain. Interestingly, while AP2 domains are found in a wide range of plants and even some bacteria without a CRF domain, the CRF domain is never found in any protein without an AP2 domain [3,6]. Proteins that contain the CRF domain make up about 10% of Arabidopsis ERF proteins (12/122), 6.5% of rice ERF proteins (9/137), and 6.5% of Populus (11/168) [2,5,8]. Additional estimates of the CRF domain abundance in other plant species are difficult to make without a large scale study of ERF proteins or a sequenced genome, but numbers appear to reside in a similar range of 5-10% of ERF proteins.

The CRF proteins appear to be unique to land plants. Despite the availability of several fully sequenced green algal genomes, CRF domain containing genes could not be identified in this lineage. In searching for such, we assumed that moss and bryophyte CRF domain sequences would make the best BLAST queries, although even those may have not been similar enough to any algal CRF domains to get a hit. AP2 domain containing genes are present in green algal genomes but examination of several of these [15] did not reveal any ERF type genes in these organisms.

Flowering plant CRF proteins roughly fall into two large clades, A and B, according to distance and parsimony analyses (Figure 2). That diverse species of flowering plants i.e. both monocots and eudicots, occur in each of these clades suggests a relatively early divergence of the two CRF lineages.

Clade A contains approximately twice as many CRF genes for numerous plant species as the B clade. Previous examinations of then unknown CRF proteins from larger scale analyses of the entire ERF protein family, have hinted at such a discrepancy for some species but lacked resolution of clade A and B members [2,5,8]. Our analysis of a wide range of species strongly suggests that B clade CRFs, while slightly divergent from A clade members, clearly belong to the CRF group and not any other ERF sub-group.

An additional motif found in a number of CRF proteins is the amino acid sequence SP [T/V]SVL in the C-terminal end of the protein downstream of the AP2 domain. Although this motif is found in all of the previously described Arabidopsis CRF genes, similar genes from rice (subgroup VI), and both Tomato Pti6 and Tsi1 it is not ubiquitous among CRF proteins occurring in roughly half of the CRFs identified here for which full length sequence is available [8,12,13]. Additionally, in contrast to the CRF domain, a SP [T/V]SVL motif is known to occur in a number of proteins outside of the AP2/ERF family, including Phosphatididylinositol transferases, Universal stress proteins, LRR/extensions, and Myb and Zinc Finger transcription factors. There are 33 non-CRF proteins that fall into this category in Arabidopsis alone. Interestingly, several of the members of this group have also been shown to have effects on leaf development like the CRF genes, including Early Phytochrome Responsive1 (EPR1), Longifolia (LNG1 and LNG2) and Growth Regulating Factor3 (GRF3) (Additional File 2). While the effects of leaf development vary among this group it is revealing that nearly 30% of Arabidopsis proteins with this motif can be linked to leaf development. Perhaps more significant is the apparent link between this motif and regulation by the hormone cytokinin, as nearly half of the non-CRF genes (14 of 30) that contain this motif and have been examined on Affymetrix microarray experiments, show altered transcription levels by treatment with cytokinin or in cytokinin mutants (Additional File 2). It is possible that the SP [T/V]SVL motif functions as a putative MAP kinase and/or casein kinase 1 phosphorylation site, as part of this motif has previously been described as such [8,9]. Such a function may serve to link CRFs to cytokinin regulation as phosphorylation is an essential part of other members of the cytokinin signaling pathway [16].

It is currently unclear as to the exact function of CRF proteins. Only eight have been experimentally examined prior to this report: CRF1-6 from Arabidopsis, PTI6 from Tomato, and TSI1 from Tobacco. While all CRFs possess an AP2/ERF binding domain and are most closely related to Ethylene Response Factor (ERF) proteins directly involved in ethylene response, there is no evidence that CRFs are linked to ethylene save the putative ability of their AP2 domain to bind to the ethylene response element, GCCGCC. None of the Arabidopsis CRF genes show any transcriptional change in response to ethylene or in ethylene mutant backgrounds in microarray experiments. Additionally, CRF1-6 mutants bear little resemblance to classic ethylene mutant phenotypes and show no sign of variation in ethylene levels, even in analysis of triple mutant knockout lines [[17], Rashotte and Kieber, unpublished result].

One potential role of CRF genes could be in cytokinin regulation as all six Arabidopsis CRF proteins examined have been shown to be regulated by cytokinin in terms of their intracellular, particulary nuclear localization - presumably the site of action for these transcription factors [10]. However, only three of these six CRF genes, CRF2, CRF5, and CRF6 are regulated by cytokinin transcriptionally and the other three do not show any transcriptional regulation by cytokinin as examined in microarray experiments [10,18,19]. Future study at the protein level has yet to determine if the other Arabidopsis CRF proteins are similarly cytokinin regulated, and none of the newly discovered CRF proteins identified in other plant species here have been examined in this manner to date.

Only one study prior to this one has examined cytokinin regulation at the transcriptional level outside of Arabidopsis, Hirose et al., 2007 [20]. This study in rice used microarrays to examine global expression patterns of genes regulated by cytokinin application and in a cytokinin response regulator overexpressing plant. While this work did identify several highly related ERF family genes that are induced by cytokinin similar to the CRFs, and supports the general role of ERFs in cytokinin regulated processes, the induced ERF genes in rice do not contain either a CRF domain or a SP(T/V)SVL motif in their protein sequences [20]. The cytokinin induced ERF genes are related to CRFs, but lack CRF domains and have been placed by sequence analyses into different subgroups after careful examination (group B-3 or VII vs. group B-5 or VI [2,8,20]). Further examination of the other members of this group and of just the CRF domain alone are needed to determine if this domain is specifically involved in cytokinin regulation of these proteins. Despite the current lack of evidence it is an attractive hypothesis that the CRF domain is somehow involved in cytokinin regulation.

Mutational analysis of CRF genes is likewise limited to studies in Arabidopsis although a RNAi knockout of Tsi1 was generated in tobacco [10,21]. The study of mutants in CRF1 to CRF6 genes has indicated a possible role in cotyledon, leaf, and embryo development in addition to their link to cytokinin [10]. There are no specific reports of additional phenotype alterations in any of the other Arabidopsis genes containing a CRF domain or in any other species to date, although this is may be due to either a lack of mutants, lack of study, or potentially redundant nature of CRF genes as seen in Arabidopsis.

In order to further examine if cytokinin does play role in the regulation of newly 'discovered' CRF proteins outside of Arabidopsis we examined four CRF genes identified in this study from Tomato. We chose these Tomato genes as it allowed for the examination of both a known CRF protein, PTI6 that we also designate SlCRF1, and three novel proteins, SlCRF3-5, none of which had previously been examined for cytokinin or any other response. An analysis of SlCRF transcripts in Tomato leaves in the presence and absence of cytokinin treatment shows that all four SlCRFs are induced by cytokinin to varying degrees (Figure 3). This significant result shows that SlCRFs are truly regulated by cytokinin, at least at the transcriptional level, and suggests that CRF proteins may generally play a role in cytokinin regulation. It is interesting that all the SlCRFs show transcriptional cytokinin regulation, when not all Arabidopsis CRFs are known to be transcriptionally regulated, especially since all CRF domain containing genes in rice appear to not show this regulation as seen in one microarray examination [10,20].

Additional putative functions for CRF proteins including links to pathogen response and salt stress come from the two other CRF proteins that have been previously examined in some detail for function, Pti6 from Tomato and Tsi1 from Tobacco [12,13]. Pti proteins Pti4/5/6 are known to interact with Pto Kinase that is directly linked to pathogen response, as this is how they were originally identified [12]. All three of these Pti proteins are also ERF proteins, yet only Pti6 can be classified as a CRF protein. While it is unclear how these Pti proteins act with Pto kinase in pathogen response, it appears that the CRF domain is not essential of that interaction. Not only do both Pti4 and 5 lack that domain, but an examination of the yeast-two hybrid analyses to determine what parts of the Pti proteins are required to interact with the Pto kinase showed that the initial 48 amino acids of Pti6, containing at least half of its CRF domain, was not necessary [12]. Tsi1 has been linked by transcript induction to high salt stress and bacterial pathogen resistance in 35S overexpressing Tsi1 transgenic plants and to similar stresses in Tsi1:RNAi plants [13,21]. Interestingly, Park et al., 2001 also found that the AP2 domain of Tsi1 was able to bind both the GCC box involved in ethylene response and also the CBF/DREB cis-element involved in drought stress response. This would suggest the involvement of the AP2 domain in the salt stress response of Tsi1. Together these analyses of Pti6 and Tsi1 clearly indicate a role of these proteins in pathogen response. This suggests that pathogen response may be a larger function for the CRF group of proteins, although there is little evidence to suggest any CRF proteins other than Pti6 and Tsi1 are involved in pathogen responses as evidenced from the lack of response in several pathogen response microarray analyses of Arabidopsis CRF genes. Response to salt stress could also a part of CRF protein function as Tsi1 has been shown to function in that area and there is some microarray data that a few Arabidopsis CRF genes could be involved, but little is known beyond that. A more detailed analysis of pathogen and salt stress responses will have to be made before any clear function can be ascribed to this group of proteins or genes, but as members the members of this group are now defined it should be easier to compare and compile these and other potential functions in the future.

## Conclusions

We have defined a distinct subgroup of AP2/ERF proteins comprised of over 125 members from a wide range of land plants that we designate as CRF proteins. These proteins can be specifically characterized by a conserved, novel domain unique to this group, designated as the CRF domain, which is always found in a N-terminal position to an additionally CRF-specific AP2 DNA binding domain. Many CRF proteins also contain a putative phosphorylation motif in their C-terminal end. Distance and parsimony analyses of the CRF and AP2 domain sequences of these proteins suggest that there are two major lineages of flowering plant CRFs, here designated clade A and B. Additional support for the distinction of these two clades is provided by the presence of the TEH region, an N-terminal extension of the CRF domain that is found only in clade A proteins. There are roughly twice as many CRF clade A sequences as clade B available in databases and the same 2:1 ratio is seen in several fully sequenced genomes.

Only limited functional analyses have been performed on the eight previously known CRF proteins, those suggesting potential roles in cytokinin regulation, pathogen response or salt stress. An examination of four newly identified CRFs from Tomato (SlCRFs) revealed that they are also induced by cytokinin, further supporting the idea that CRF proteins may function in general in cytokinin regulation. Additional work on more CRF proteins is needed to determine the overarching function of this group.

## Methods

### Database searches

Putative CRF genes were identified in sequence databases with BLAST searches using blastx, tblastn and tblastx. Additionally, iterated profile searches were conducted using PSI-BLAST (Position-Specific Iterated BLAST). CRF, TEH, and AP2 domains from diverse CRF genes (as they were discovered), and variously broad consensus sequences of each of these domains were used as query sequences in multiple searches. All sequence databases were also queried using HMMER3 software that employs hidden Markov probabilistic models capable of detecting remote homologs [22]. Default settings of the various BLAST programs were used except for the low complexity filter and compositional adjustments. In searches for divergent homologues, the statistical significance threshold for reporting matches was raised, word size decreased to 2, and matrix and gap costs varied. NCBI NR/NT, EST, GSS, HTGS and WGS databases as well as trace archives and several (not yet NCBI accessioned) ongoing sequencing project databases were searched for CRF domain proteins. To identify putative CRF genes belonging to the earliest branching lineages of land plants, BLAST searches were performed in an iterative fashion i.e. successively 'deeper' hits representing earlier branching lineages were used as queries in five additional round of searches.

### Sequence analyses

Motif detection was performed with MEME (MEME version 4.1.0, http://meme.sdsc.edu/meme/) [23].

All CRF domain containing proteins were aligned across their TEH, CRF, AP2 domains and 3' regions where possible. Amino acid alignments were performed in ClustalX 2.0 using all default options with manual adjustments where necessary. Only CRF and AP2 domain regions were included in phylogenetic analyses, and the final alignment of 136 sequences contained 85 characters, alignment available from the authors upon request.

Phylogenetic analyses were performed in PAUP* v.4.0b11 [24] using both the Neighbor Joining method and also Maximum Parsimony (MP) as an optimality criterion for tree evaluation. The heuristic search strategy involved thousands of random sequence addition replicates with tree bisection-reconnection (TBR) branch swapping. To assess support for each node, bootstrap analyses [25] were performed using 1000 pseuodreplicates and 10 random addition replicates with TBR branch swapping within each bootstrap replicate. Specific Genebank accessions reference for species labels in Figure 2 are shown in Additional File 1.

### Examination of SlCRF gene transcripts

Tomato seedlings (*Solanum lycopersicum *v. moneymaker) were germinated in Sunshine Mix #8, initially grown for 10 days in a growth chamber at 16 h light at 22C and 8 h dark at 18C and then transferred to a greenhouse maintained at 24C. To examine cytokinin regulation, leaves from a 20 day old plant were excised and placed in water and gently shaken for 1 hour at which point either 5 μM cytokinin (benzyladenine) or the carrier solvent DMSO was added with shaking allowed to continue (after Rashotte et al., 2003[19]). After a 2 hour treatment leaves were patted dry and RNA was extracted by flash freezing and grinding tissue in liquid nitrogen prior to extraction using a Qiagen RNeasy kit. RT-PCR (25 cycles) was performed using this tissue-derived RNA via a Qiagen One Step RT-PCR kit with gene specific primers for each SlCRF and beta-tubulin. This was repeated on three biological samples, with a representative outcome shown in Figure 3. Primers are as follows. SlCRF1 (SGN-U579886, PTI6) forward 5' GGAAAATTCAGTTCCGGTGA 3', reverse 5' AAAATTGGTAACGGCGTCAG 3'. SlCRF3 (SGN-U573201) forward 5' AATGATGCAGTCGAGGAACC 3', reverse 5' CCTGGTCTTCCCATTCTCAA 3'. SlCRF4 (SGN-U574151) forward 5' TGAATCCCTCTGTTCCAAGG 3', reverse 5' GTTTTGCCATTTCCACTGCT 3'. SlCRF5 (SGN-U583231) forward 5' ACGATGACGACGAGAGGAAT 3', reverse 5' CTGACACCGCGAAACTTTTT 3'. Beta-tubulin (SGN-U564000) forward 5' ATTCCAGGTTTGCCACTCAC 3', reverse 5' GCTTCGTTGTCAAGGACCAT 3'.

## Authors' contributions

AMR carried out the examination of SlCRF transcripts and helped gather the initial CRF sequence data. LRG identified and aligned the new CRF sequences and performed the phylogenetic analyses. Both authors wrote and prepared the manuscript and have approved the final version.

## Supplementary Material

Additional file 1 Taxon table of species names and GeneBank accession or reference names. Click here for file

Additional file 2 Table of Arabidopsis proteins containing the motif SP [T/V]SVL. Click here for file
