# Supplementary material for: The CRF domain defines Cytokinin Response Factor proteins in plants
Source: BMC Plant Biol. 2010 Apr 26;10:74. doi: 10.1186/1471-2229-10-74 (PMC3095348; doi:10.1186/1471-2229-10-74)
Supplement: Additional file 1 — Taxon table of species names and GeneBank accession or reference names. [file 1471-2229-10-74-S1.PDF]

tree label	Genbank accession or reference	
Actinidia	FG489491	
Allium	CF450138	
Aquilegia1	DT728900	
Aquilegia2	DR928090	
Arabidopsis CRF1	At4g11140	
Arabidopsis CRF2	At4g23750	
Arabidopsis CRF7	At1g22985	
Arabidopsis CRF8	At1g71130	
Arabidopsis CRF3	At5g53290	
Arabidopsis CRF4	At4g27950	
Arabidopsis CRF5	At2g46310	
Arabidopsis CRF6	At3g61630	
ArabidopsisB1	AY560840	
ArabidopsisB2	BT005281	
ArabidopsisB3	AT1G68550	
ArabidopsisB4	AT3G25890	
Artemisia2	EY106320	
Artemisia1	EY085477	
Asparagus	CV292627	
Beta	BQ591872	
Brassica1	EE468669	
Brassica5	ES921419	
Brassica2	EE424085	
Brassica3	ES941324	
Brassica4	AC189358	
Brassica6	EX104130	
Bruguiera	BP945821	
Capsicum	CA524595	
Centaurea1	EH768043	
Centaurea2	EH710818	
Ceratopteris	BE643041	
Cichorium1	EL345647	
Cichorium2	EL351160	
Citrus1	DY300688	
Citrus3	CX304599	
Citrus2	DY293431	
Citrus4	DY286871	
Citrus5	CX299594	
Coffea1	DV692675	
Coffea2	DV672119	
Cucumis	AM741737	
Curcuma	DY390981	
Euphorbia1	TA12667 3993	
Euphorbia2	DV135082	
Fragaria	DY671470	
Ginkgo	EX936291	
Glycine1	BM309574	
Glycine2	TA71688_3847	
Glycine3	BI469021	
Gossypium2	DW507848	
Gossypium1	DR457764	
Gossypium3	DT563107	
Gossypium4	DT554469	
Helianthus1	EL480438	
Helianthus3	EL444901	
Helianthus2	EL420093	
Hordeum	BY836368	
Humulus	EX519353	
Ipomoea1	CJ742230	
Ipomoea2	CJ749041	
Ipomoea3	EF192423	
Lactuca1	DW059258	
Lactuca2	DY973784	
Lotus1	BW619808	
Lotus2	AP004902	
Malus1	CN888811	
Malus2	DY671470	
Manihot	CK651291	
Marchantia	BJ848446	
Medicago2	AJ503842	
Medicago3	AW685524	
Medicago1	BG646896	
Musa	ES431700	
Nicotiana1	AF058827	
Nicotiana2	CK294616	
Ocimum	DY333371	
Opuntia	DQ914857	
Oryzax1	Os01g0224100	
Oryza_ERF56	Os05g25260	
Oryza_ERF55	Os06g06540	
OsERF53	Os01g12440	
Oryza_ERF54	Os01g46870	
Oryza1	NM 1048985	Os01g0224100
Oryza2	NM 1058221	Os03g0815800
Oryza_sERF57	Os07g12510	
Oryza_ERF58	Os03g60120	
Oryza_ERF108	Os01g04020	
Oryza3	CT831165	
Oryza_ERF109	Os09g13940	
Oryza_ERF138	Os08g27220	
Petunia	CV300211	
Physcomitrella1	BY988607	
Physcomitrella2	XM_001780978	
Physcomitrella3	XM_001756051	
Physcomitrella4	XM_001753142	
Picea1	EX439121	
Picea2	CO489679	
Pinus	CF394914	
Populusx1	AC208094	
Populusx2	AC196813	
Populusx3	CK112320	
Prunus	CV044509	
PopulusPtERF-B5-1		
PopulusPtERF-B5-2		
PopulusPtERF-B5-3		
PopulusPtERF-B5-4		
PopulusPtERF-B5-5		
PopulusPtERF-B5-6		
PopulusPtERF-B5-7		
PopulusPtERF-B5-8		
PopulusPtERF-B6-12		
PopulusPtERF-B6-13		
PopulusPtERF-B6-14		
Pyrus	DV440808	
Raphanus1	EW732842_EW737698	
Raphanus2	EV565848	
Ricinus2	EG679827	
Ricinus1	EEF49000	
Saccharum	CA198236	
Saruma	DT580954	
Selaginella2	unpub	
Selaginella1	FE445691	
Solanum_ SlCRF2	U345203	
Solanum10	CK245775	
Solanum_SlCRF5	SGNU329134	
Solanum_ SlCRF1(PTI6)	SGNU314347	
Solanum_ SlCRF4	SGNU331355	
Solanum_ SlCRF3	SGNU344182	
Solanum_SlCRF6	SL_MboI0034O03_SP6_280529	
Solanum9	SGNU277128	
Solanum8	AK247850	
Suaeda	BE231371	
Trifolium1	BB936292	
Trifolium2	BB912206	
Triphysaria	EY152518	
Triticum	CD930160	
Vigna	FG886590	
Vitis1	GSVIVP00002468001	
Vitis3	VV78X091360.7	
Vitis2	VV78X041419.2	
Vitis4	AM429482	VV78X245375.21
Vitis5	AM473189	VV78X194508.12
Vitis6	XM_002267364	LOC100248494
Vitis7	VV78X085330.6	AM466649
Vitis8	XM_002271490	LOC100263984
Vitis9	XM_002279221	LOC100256010
Vitis10	XM_002276038	LOC100257771
Zea	EC888931	
Zea1	BI233683	
Zea2	DN222126	
Zingiber2	DY363423	
Zingiber1	DY358563	
