# Supplementary material for: The CRF domain defines Cytokinin Response Factor proteins in plants
Source: BMC Plant Biol. 2010 Apr 26;10:74. doi: 10.1186/1471-2229-10-74 (PMC3095348; doi:10.1186/1471-2229-10-74)
Supplement: Additional file 2 — Table of Arabidopsis proteins containing the motif SP [T/V]SVL. [file 1471-2229-10-74-S2.DOC]

**Additional File 2.** Arabidopsis proteins containing the motif SP[T/V]SVL. Proteins are listed by their ATG identifier, description of function, and matching sequence. Additional links of protein function to leaf development or two fold transcriptional change by cytokinin or in a cytokinin mutant background are noted in parentheses as L, C, or ND for no transcriptional data respectively. Matches to multiple isoforms of the same protein are not listed.

AT1G08010.1 Zinc Finger TF AtGATA-6 SPVSVL

AT1G18330.1 MYB TF EPR1(Early Phytochrome Response1) SPTSVL (L,C)

AT1G18620.1 Similar to LONGIFOLIA SPVSVL (L,C)

AT1G54130.1 RSH3(RELA/SPOT Homolog3) SPVSVL (C)

AT1G63670.1 Phosphatidylinositol

N-Acetylgucosminyltransferase subunit P(PIG-P) SPVSVL

AT1G74870.1 Zinc Finger: RING type TF SPTSVL (C)

AT2G20240.1 PIG-P SPVSVL (C)

AT2G28340.1 Zinc Finger TF GATA7 SPVSVL (ND)

AT2G36400.1 AtGRF (Growth Regulating Factor3) SPTSVL (L)

AT2G36420.1 Similar to 60S ribosomal protein L17 SPVSVL (ND)

AT2G39440.1 Unknown SPVSVL

AT2G45900.1 PIG-P SPVSVL

AT2G46310.1 CRF5 SPTSVL (L,C)

AT3G02170.1 LNG2 (LONGIFOLIA2) SPVSVL (L,C)

AT3G10113.1 Similar to EPR1 (MYB131 TF) SPTSVL (ND)

AT3G22800.1 LLR/Extensin SPVSVL (C)

AT3G24050.1 GATA TF (GATA1) SPVSVL

AT3G53540.1 PIG-P SPVSVL

AT3G54810.1 BME3 (Blue Micropylar End3)-GATA TF SPVSVL (C)

AT3G59630.1 Similar to DPH2 Diphthamide synthase SPTSVL (C)

AT3G61380.1 PIG-P SPVSVL

AT3G61630.1 CRF6 SPTSVL (L,C)

AT3G63430.1 Unknown SPVSVL (ND)

AT4G11140.1 CRF1 SPVSVL (L,C)

AT4G14605.1 Pigment Defective191 SPVSVL

AT4G23750.1 CRF2 SPVSVL

AT4G27320.1 Universal Stress Pro(USP)-CHP-Zinc Finger TF SPTSVL

AT4G27950.1 CRF4 SPTSVL( L)

AT5G01370.1 Unknown SPVSVL

AT5G03670.1 Unknown SPVSVL

AT5G15580.1 LNG1 (LONGIFOLIA1) SPVSVL (L,C)

AT5G17300.1 MYB TF SPTSVL (C)

AT5G25550.1 LRR/Extensin PEX1 SPVSVL (C)

AT5G37260.1 CIR1 (Circadian1)-MYB TF SPTSVL (C)

AT5G46740.1 UBP21 (Ubiquitin Specific Protease 21) SPVSVL (C)

AT5G53290.1 CRF3 SPTSVL (L)

AT5G54430.1 USP SPTSVL

AT5G58630.1 Glycosyl hydrolase domain containing Pro. SPVSVL (C)

AT5G66750.1 DDM1 (Decreased DNA Methylation 1) SPTSVL
